# General Anatomy. The Doctrine of the Elementary Composition and Structure of the Human Body

**Published:** 1842-10

**Authors:** 


### ART. XV.

1. *Allgemeine Anatomie. Lehre von den Mischungs-und Formbestandtheilen des menschlichen Körpers.* Von J. HENLE.—*Leipzig*, 1841. 8vo, pp. 1032. Mit funf Tafeln, &c.

*General Anatomy. The doctrine of the elementary composition and structure of the Human Body.* By J. HENLE.—*Leipzic*, 1841. 8vo, pp. 1032. With Five Plates, &c.

2. *Handbuch der allgemeinen Anatomie des Menschen und der Haussäugethiere.* Von FR. GERBER, Professor der Thierheilkunde, &c. in Bern.—*Bern*, 1840. 8vo, pp. 213. Mit 7 Steindrucktafeln in Folio.

*Manual of the General Anatomy of Man and the Domestic Mammalia.* By FR. GERBER, Professor of Veterinary Medicine, &c. at Bern.—*Bern*, 1840. 8vo, pp. 213. With 7 Lithographic Plates in Folio.

3. *Elements of the General and Minute Anatomy of Man and the Mammalia.* By FR. GERBER. *To which are added Notes and an Appendix, by* GEORGE GULLIVER, F.R.S.—*London*, 1842. 8vo, pp. 390-106. With an Atlas, containing 34 Plates.—*London*, 1842. pp. 71.

4. *Lehrbuch der allgemeinen Anatomie des Menschen.* Von VICTOR BRUNS, Doctor der Medicin und Chirurgie, &c.—*Braunschweig*, 1841. 8vo, pp. 398.

*Elements of the General Anatomy of Man.* By VICTOR BRUNS, Doctor of Medicine and Surgery &c.—*Brunswick*, 1841.

5. *Die Mikroskopischen Forschungen im Gebiete der menschlichen Physiologie, dargestellt von* OTTO KÖSTLIN.—*Stuttgart*, 1840. 8vo, pp. 304.

*The Microscopic Researches in the department of Human Physiology, set forth by* OTTO KÖSTLIN.—*Stuttgart*, 1840.

6. *Traité pratique de Microscope et de son emploi dans l'étude des corps organisés.* Par le Docteur L. MANDL; *suivi de Recherches sur l'Organisation des Animaux Infusoires.* Par Dr. D. C. G. EHRENBERG. *Paris*, 1839. 8vo, pp. 486. Avec quatorze Planches.

*Practical Treatise on the Microscope, and its employment in the study of organized bodies.* By Dr. L. MANDL; *followed by Researches on the Organization of Infusory Animals.* By D. C. G. EHRENBERG.—*Paris*, 1839. With Fourteen Plates.

7. *Anatomie Microscopique.* Par le Docteur L. MANDL. Parts I-V. —*Paris*, 1838-9. Fol. maj.

*Microscopic Anatomy.* By Dr. L. MANDL. Parts I-V.—*Paris*, 1838-9.

8. *Entwurf eines neuen genetischen Systems der Histologie, zugleich als Grundriss einer philosophischen Anatomie.* Von Dr. HERMANN KLENCKE.—*Leipzig*, 1841. 12mo, pp. 230.

*Attempt at a new genetic system of Histology, for a basis to a philosophical Anatomy.* By Dr. HERMANN KLENCKE.—*Leipzic*, 1841.

9. *On the Employment of the Microscope in Medical Studies. A Lecture introductory to a course of Histiology.* By JOHN HUGHES BENNETT, M.D.—*Edinburgh*, 1841. 8vo, pp. 27.

I. The report on minute anatomy which we lately published, renders it unnecessary to analyse the contents of the works whose titles are prefixed. Our present intention is to speak briefly of the characteristics of each of them, and to offer some remarks on the history of their common subject, and on the spirit and method in which it is now investigated.

1. The work of Professor Henle is a complete system of general anatomy, and such an one as probably no other man in Europe could have written. Its author is already known to our readers as one of the most sharp-sighted and industrious anatomists of Germany; as the most successful investigator of the nature of mucus, and of the structure and arrangement of the epithelia, the hairs, the blood-vessels, the villi, and the true elementary parts of the secernent glands. By his discoveries in these and many other particular objects of inquiry, he has contributed to the knowledge of physiology scarcely less than his fellow-pupil, Schwann, has by his great generalization of development through cells. For the present work Henle seems to have laboured in the whole field of structural anatomy both by actual investigation and by reading; and he has thus produced a book which is not only superior to all others on the same subject, but has few equals in merit among the text-books of other sciences. It is written honestly, accurately, and with common sense, and, though far too large for the use of students, it would be hard to say what part could be omitted without lessening its value to those who are already acquainted with the general principles of the science.

Of its two chief sections the first, on the chemistry of the body, is compiled from the works of Berzelius, Löwig, and F. Simon; the second, which forms nine tenths of the whole, is devoted to structural anatomy. There is in it a happy combination without confusion of the facts of observation with those of history. In each chapter, a tissue is first described with no more references than are absolutely necessary, and this is followed by the history of the progressive discoveries in its anatomy, and an analysis of the opinions of different authors on questions yet unsettled. In all this historical part, Henle's accuracy and impartiality are truly admirable: we have scrupulously compared page after page with extracts from the quoted works and have hardly discerned an error.

2. The value of Dr. Gerber's Manual, which at its first appearance was the best of the modern works on general anatomy, has been greatly depreciated by the publication of those of Henle and Bruns. Not that it was admirable even when almost unrivalled; for its style is obscure, and the descriptions of all the tissues are confused or imperfect. It contains, indeed, many original facts from the investigations of its author and his friend Valentin; and these give it considerable value. But its general usefulness is prevented by its faults, not only of style, but of arrangement; the author seeming to have mistaken minuteness of division for accuracy of plan.

Of the translation the best that can be said is that it holds in English literature the same place which the original work once did abroad; it is unrivalled; it is executed with a fair amount of accuracy, but the writer seems often to have stuck half-way between the languages, and the translation of many words has to be carried some distance further before they are fairly rendered into English. The plates are, in both, hard and unnatural: they are, indeed, little more than diagrams of the objects they are intended to represent. Much value is added to the translation by Mr. Gulliver's notes and appendix, in which he has embodied the results of his laborious investigations of the blood-globules, the chyle, lymph, &c.

3. The characters of Bruns' work are equally different from those of both the preceding. It contains but little original matter, but its descriptions are clear, brief, and, according to the generally received views, complete; no extraneous questions are introduced, and the simple tale is told as fluently as if it were all true and fit to be applied to any extent in practice. By all these qualities it is as well adapted for the use of the student as Henle's work is for that of the professor, or of one who is already acquainted with all that Bruns could tell him.

4. Otto Köstlin's book is a compilation of abstracts of all that, at the time of its publication, had been learnt by the use of the microscope, and merits nearly all the praise that can be given to success in such an undertaking; for it is complete and accurate. There are very few parts in which the author has either omitted or misunderstood the labours of those who have worked on the subject. Its defects are the attempts to graft ideal physiology upon substantial anatomy. It is hard to conceive how a man, having learnt so many facts, could believe that there is anything but nonsense in the notions which he tries to make them illustrate. If he had wished to prove how little connexion there is between the two things, he could not have succeeded better than by thus merely putting them side by side.

5. The treatise of Dr. Mandl is the best which has been produced in France upon microscopic anatomy. But it is inferior to all the preceding. It contains a well-written history of the microscope; but the few pages which follow on some of the tissues are imperfect, and, in the present state of microscopic science, valueless. The whole style of the work is too light for a subject which, at present, consists of little more than facts. The value of the researches of Ehrenberg is well known: but Dr. Mandl has been unwise to add them to his work, for they not only have no natural connexion with what precedes them, but, by their completeness, they render the imperfections of the rest more obvious.

6. The Anatomie Microscopique of the same author is scarcely more than an enlarged and better illustrated edition of the treatise. It includes only the tissues which Dr. Mandl has particularly studied; but his investigations fall far short of the accuracy and completeness of the German anatomists. The plates have the faults common to so many of those intended to represent microscopic objects; in the attempt to make them distinct, they are made stiff, diagram-like, and too definite. Nothing but the rare association of an artist who is an anatomist, with an engraver of the first degree of excellence, can ensure an accurate representation of nature in works of this class; and in none of those before us has such a combination of talents been employed. Indeed we know not where they are to be found in the service of general anatomy, except in the works of Berres and Wagner.

7. The title of Dr. Klencke's work at once reveals something of its character. It is one of those confident attempts which are commonly made in Germany to establish an imaginative system, in the notion that it is the offspring of the pure reason. The author says of it in another book,* that Carus has honoured it by the assertion that, " by this work the histotomy of former days has been for the first time carried out to a real histology." Our readers may judge how far it has effected an advance in science by the following extracts, which it is necessary to give as an example of the style to which we shall have again to refer. As one of the principles on which physiology is to be placed on an imperturbable basis he sets down this;

"Since every formation is the plastic expression of the vital-idea working in it, and this idea is, in every organism, a focal point of the general universal-idea, and this universal-idea is the very Godhead impressed in nature; it follows that the world and all forming existence must go on with the completest reason and adaptation to purpose; and there must be manifested in every phenomenon a thinking which points back to reason. There lives, then, in all nature a thinking without consciousness, a dreaming intelligence, which is alluded to by natural philosophers as a *nisus formativus*, instinct, &c. But in man this thinking bursts into consciousness; and the same idea which is formative in his organism, and, without being the subject of consciousness, unfolds the living members from the germ, itself becomes perceptible; and now, since in itself it represents the universe, it reflects as from a mirror, in the light of the conscious reason, all nature and the eternal foundation-thought of the macrocosm; and it inflames spiritually the general intelligence in the realm of spirits. Consciousness, and unconscious organizing are therefore *one* undivided and idealized principle." (p. 15.)

In other words, and this might be the first article in the creed of this sect, " thinking is knowledge." Another passage will show the kind of knowledge attained by this employment of the pure reason in questions in the solution of which the understanding has its proper work.

"What," asks the author, "is this seemingly watery substance with which all the tissues are moistened?—and he answers in the words of his masters, Carus, who calls it the 'parenchymatous primary formative fluid,' and Oken, who says, 'It is the true nervous substance.' With the nervous matter has the animal substance commenced; with that which is the highest, and which physiologists have regarded as the last. The origin of the animal is from nerve, and all anatomical systems are only unwindings or expansions from the nervous mass. The animal is nothing but nerve; what it is further is nerve-metamorphosis. The mucus of the medusæ and polyps is nerve-substance at the lowest stage, in which the inherent and confused substances are not yet developed in isolation. The nervous matter is the absolute Indifferent in the animal; that which by the lightest breath, even by a thought, is polarizable." (Klencke, p. 44.)

8. Dr. Bennett's Introductory Lecture is an unconditional, and therefore an unwise, panegyric on the microscope. All that is stated on microscopic evidence is advanced as if it were certain truth; yet in the lapse of a year doubt has been cast on a great moiety of the *facts* which it contains. It includes, however, some interesting cases, in which the microscope afforded assistance in the diagnosis of disease.

II. Having said thus much of these works, of which the mere existence is a remarkable evidence of the advance and progress of the anatomy of structure, let us now examine briefly the stages through which it has passed in its course hitherward; an inquiry which, as far as we know, has not hitherto been made, and which, by directing it chiefly towards that part

* Neue Anat. und Phys. Untersuchungen über die Primitivnervenfasern und das Wesen der Innervation, p. xl.

of anatomy which is studied by the microscope, may serve to illustrate some of the faults of the spirit in which it is pursued.

The beginning of the study of structural anatomy was probably coincident with the first strict inquiries into the form and nature of the human body. As soon as men began to examine how the parts of their frame were put together, they would naturally investigate also the structure of each part; and, with scarcely an exception, we find that, to the time of Bichat, the anatomy of structure and that of construction were treated as one science. The latter made, as may be supposed, by far the more striking and rapid progress; and it is not possible to trace any stages of discovery in structural anatomy till the time of Malpighi, who first, about the year 1660, employed magnifying glasses in the study. The first fruits of microscopic research were rich and abundant. Not to mention Malpighi's discoveries in vegetable physiology; the discovery of corpuscles in the blood, and of the capillary circulation, the main fact of the cellular structure of the lungs, and the principles of the formation of secernent glands, the import of the sensitive papillæ, the lobular and cellular structure of the fat, the mode of the growth of hair, as much as can be believed of the structure of the spleen, the more important part of the anatomy of the kidney, the threefold constituents of the teeth, and many facts of less interest were all fully established by him, and to all the microscope was an important, if not an essential, aid. It is remarkable that Malpighi's were almost the only facts discerned in the investigations of the first microscopic anatomists which stood the test of time. Though often denied, yet scarcely one of them has been even temporarily obscured; they were at once established as truths, or as very probable opinions; and no change of doctrine could remove them from the accepted records of medical science. The explanation of so striking a fact is obviously this; Malpighi reasoned rightly, or but little on his facts, and, possessing a thorough knowledge of the physiology of his day, he was able at once to point out many of the relations which his discoveries bore in the explanation of processes in which all were interested. In this he was widely distinguished from those who immediately followed him in similar investigations, and of whom scarcely one knew more of physiology than of many other sciences. It is, indeed, difficult to speak too highly of the merits of Malpighi. He was in every respect a great physiologist; and in the history of microscopic anatomy he stands pre-eminently among the few who have combined acuteness in minute researches with clearness of reasoning upon the results which he obtained.

After Malpighi, the next great name in the history of minute anatomy is Leeuwenhoeck; whose earliest investigations appeared in 1673, in a communication made to our Royal Society through Regner de Graaf. There is a striking contrast between the two; Malpighi, we have said, was a genuine and accomplished physiologist; Leeuwenhoeck was an unlettered mere microscopist; the one used his microscope with a truly philosophic view, the other's microscope may be said rather to have used him. Leeuwenhoeck looked at everything that was small enough to escape the eyes of others; without order, and as if without purpose, he examined objects from every source. His instruments and his eye were, however, alike marvellously accurate; and employing them with extraordinary industry, he was enabled to perfect and discover more facts than perhaps any other anatomist of any time, so that it is still hard to find any thing which he has not discerned, and it is unwise of any man to appropriate a discovery with the microscope until he has assured himself that Leeuwenhoeck has not a prior claim. No man of his day would have been greater, had he been able to make a scientific use of all the facts that he collected; but unhappily, when he left the regions of sense he almost always went into those of error; his imagination was apt to see in every wonder something still more wonderful, in every diversity some strange and false analogy.

Among Leeuwenhoeck's discoveries in minute anatomy may be enumerated those of the bone and cartilage corpuscles, the spermatic animalcules, the tubules of dentine, and the fibres of enamel, the scales of the cuticle, and of the coarser epithelia, the chyle and milk corpuscles, the muscular fibrillæ, and the transverse striæ of their fasciculi; the tendinous and nervous fibres, and the fibrous and laminar structure of the lens and corneæ. These, and numerous other structures were seen by him, and, with few exceptions, all were described with singular accuracy. But nearly all were misapprehended; and hence it is that though Leeuwenhoeck's discoveries were ten times more numerous, and many of them were not less important than Malpighi's, yet his good influence upon science was comparatively nothing. Indeed, in later times, his frivolous and false interpretations of the structures which he saw tended to bring the microscope into disrepute; and while all Malpighi's observations had become a part of the real property of science, Leeuwenhoeck's remained unproductive for 150 years, and were rarely noticed except that the deductions from them might be laughed at. For it was not by their discoverer alone that all these facts of structure were misinterpreted. He had the misfortune to live in and before the last great period of system-making in medicine; and some of his facts, as pliant as they were striking, were admirable materials on which to found hypotheses. To what volumes of fancy did his mulberry-like blood-globules, and his globules of various orders give some semblance of truth!—All pathology, in the Boerhaavian doctrine of "error loci," seemed for a time to rest on a few of his facts, and when that pathology was proved false, the foundation was hidden beneath the fallen superstructure.

The investigations of both Malpighi and Leeuwenhoeck were made with simple lenses, and they afford a high testimony to their accuracy to the furthest limit of the field in which they can be used. Robert Hooke, the author of the "Micrographia, or physiological descriptions of minute bodies made by magnifying glasses," and the contemporary of Leeuwenhoeck, seems to have been the first who used, in the study of anatomy, the compound microscope, which about that time was invented by Jansen, or by some one even the name of whom has been forgotten. Hooke was a man of strange eccentric mind; one who spent more time in thinking than in observing, and was therefore little able to cope in discovery with the active Leeuwenhoeck. He made, however, many good observations; among them the first of the ultimate structure of muscle, which he seems to have published a year before Leeuwenhoeck.

Another contemporary, to whom minute anatomy is indebted, was John Swammerdam. Devoted to natural history, and a truly scientific naturalist, he studied structures, like Malpighi, only as the means to the knowledge of processes. His minute investigations of the anatomy of insects, to which he had been led by a taste inherited from his father, a thorough Dutch collector, was a good preliminary exercise to his more widely extended researches. At Leyden and at Paris he was the best minute dissector of his day; and his ingenuity constantly supplied him with aid when his hands and eyes were insufficient. He supplied many facts of structural anatomy, but he himself threw them into obscurity by the brilliancy of his invention of the arts of making dry inflated preparations, and of permanently injecting blood-vessels, which, with the constant assistance of Van Horne, he perfected about the year 1666.

One cannot now adequately conceive the value of such a boon as this. What weariness and labour did it spare to those who before had been content to dissect vessels filled, if at all, with air or liquids that oozed through every puncture! No wonder that the first structure whose vascularity was thus demonstrated, was called "a miracle of nature."* The beauty, as well as the importance of the structures which are thus exhibited and made permanent, was at once, as it has been ever since, a constant excitement to industry in the employment of the art. Ruysch, though he does not confess it, learned the method from Swammerdam, and he soon so far outstripped his master, that, among injectors, none has ever had so high a reputation; though, in truth, his preparations cannot bear comparison with those of Lieberkuhn, Prochaska, Barth, Sömmering, or many of our own time.

Nehemiah Grew, who lived at nearly the same time, was another who avoided the merely microscopic use of the microscope; and, had he given as much attention to animal as he did to vegetable anatomy, he must have been one of the greatest contributors to our science. As it was, we owe to him the first hints of the structure and arrangement of the perspiratory glands,† some accurate notions of the structure of the lens, and a very clear account of the intestines, in which he describes the Peyer's bodies;‡ of these last, indeed, he must be regarded as the discoverer, since his account of them was read to the Royal Society in 1676, and Peyer's inferior description of them was not published till 1677.

But before the close of the seventeenth century much of the fashion of the microscope seems to have passed away, and in the eighteenth, though anatomy was diligently cultivated, we find the facts yielded to it by the microscope scattered at very distant periods. Much of this decline is due, no doubt, to the difficulty of obtaining good instruments; for the observer had, in general, to make his own, and much of Leeuwenhoeck's success was certainly due to the superior care and dexterity with which he formed and polished his lenses; for in this, as well as in using them, he was unrivalled. But much more, probably, was due to the impossibility of making the results of microscopic researches square, as they were interpreted, with the current physiological laws and hypotheses; for by these the error of some could be detected, and suspicion could be cast on all. We say "as they were interpreted;" because, as mere facts, Leeuwenhoeck's descriptions were, through nearly all his life, notorious and popular; the number of his papers inserted in the Philosophical Trans-

* "Miraculum naturæ, seu uteri muliebris fabrica." † Phil. Trans. No. 159.
‡ The Comparative Anatomy of the Guts.—London, 1681.

actions between 1673 and 1723 sufficiently proves their notoriety; and the exaggerated praise, in which they were compared to Delphic oracles, establishes their popularity; nothing, therefore, but misinterpretation of them could make them seem valueless.

The history of minute anatomy after the death of Leeuwenhoeck may be traced in the works of a very few observers, and these not the most renowned of their day; for as contemporaries of Ruysch, Albinus, Valsalva, Morgagni, Winslow, Lieutaud, Portal, Cowper, Cheselden, and Haller, those whom we have now to mention were certainly of inferior renown.

One of the first was Wier Muys, the author of a ponderous treatise on the Muscles;* a dull man, with a great love of divisions in threes, whose discoveries bore no proportion to his researches. He seems, however, to have had a clear view of the structure of muscle, and to have been the first to examine closely the tissue of the blood-vessels, and the intermuscular fibrils. He made also several examinations of the blood-corpuscles; but, on the whole, he contributed little to the advance of science.

One of his contemporaries, J. N. Lieberkuhn, was a man of fewer words and more facts. He was the inventor of the plan of corroding preparations, about the year 1748, and as a minute injector was never equalled, unless it were by Soemmering, to whose preparations, examined in comparison with many by Lieberkuhn, Barth, and others, Prochaska† has awarded the highest praise. To Lieberkuhn we owe the first good account of the anatomy of the villi, and of the minute tubular glands of the small intestine, which still bear his name,‡ as well as of the arrangement of the blood-vessels supplying and the epithelium covering the villi.

At this time lived Ledermüller. He also looked at everything that was small; but after Leeuwenhoeck he could make few discoveries. He published numerous observations on the seminal animalcules, and there was a dispute between him and Dr. Asche, who maintained that they were mere cells, and had no tails. Recent investigations have made it probable that each had truth on his side, and that they only saw the same bodies in different stages of development. But we do not find that Ledermüller added much to the facts of science. He was content that his highest ambition should be to give others all that he had in great labour pursued, namely, "microscopic amusements for the mind and eyes," for so he called his last and longest work.§

We must not pass over Stephen Hales, the "vir pius et solers, neminique invisus," as Haller called him, who lived at this time, and whose microscopic observations, though few, were good, and were carried on in such a truly physiological spirit, as might have made many of his medical contemporaries blush. In relation to general anatomy, Hales‖ contributed some of the earliest and best experiments of the dyeing of bones by madder, observations on the blood-corpuscles, and on the velocity of the

* Musculorum artificiosa fabrica. Lugd. Bat. 1751. The first part appeared in 1738.
† Disq. anat. phys. organismi corporis humani.—Viennæ, 1812.
‡ De fabrica et actione villorum intest. tenuium.—Leid. 1745.
§ Microscopische Gemüths-und Augenergötzungen.—Nuremberg, 1763.
‖ In his Statistical Essays, vol. ii. 1740.

capillary circulation, and the account of the zig-zag contraction of the muscular fibres, which was rediscovered by Prevost and Dumas.

A contemporary ecclesiastic, Antonio della Torre, though a more laborious microscopist, accomplished less than Hales; for, like several of those already mentioned, he lacked a scientific mind to give life and force to his facts. Dr. Martin Barry (Philos. Trans. 1841) supposes that Della Torre really saw in the blood the rings which he described, (Philos. Trans. 1765, vol. lv.) and which others had mistaken, as he believed, for globules or discs; but the greater probability is, that this was but one of the many instances in which error has chanced to express itself in the language of truth; for the blood-corpuscles have central apertures only rarely, and the apertures are very hard to see: they could not have been discerned with such lenses as Della Torre used. It seems probable, however, that he saw both the ganglion-globules and the tubes in the structure of the brain; but his view of them was too obscure to add any likelihood to the supposition that he saw clearly any structure yet more minute, such as that of the blood-corpuscles.

After the middle of the eighteenth century the decline of microscopic anatomy continued to be rapid, in spite of the labours of two of the best physiologists that ever cultivated it; we mean William Hewson and Felix Fontana.

Hewson must ever be remembered for the accuracy of his observations on the blood and lymph corpuscles; and we are rejoiced to find Mr. Gulliver, who has worked with so much industry in the same field, claiming due honour for his predecessor;* for Hewson's merit as an observer has not hitherto been fairly appreciated. As far as his descriptions were carried they were almost without error; and, though the blood-corpuscles had been so often investigated, it was he who was first able to prove their flatness, (for at his time they were commonly supposed to be globular,) and the existence of a distinct central nucleus.† But he had the imprudence to append an improbable hypothesis to his facts, and they both fell into the discredit which only one deserved.

The investigations of Fontana were spread over a wider field, and many of them were singularly accurate, though at one time he seems to have had some bad glasses, which, like the celebrated lenses of Dr. Monro, made everything look like a set of spirals. Among his better observations are those of the structure of tendon, which was before nearly unknown; of the true import of the transverse striæ on the muscular fasciculi, which he first ascribed to the fibrils within the sheath; of the central substance of the nervous fibre, in which if he erred he did no more than Remak and some of the best modern observers do; and some accurate accounts of the structure of hair and of the fat-cells. But Fontana was not a mere microscopist. It has happened unfortunately for him that the only work of his which is generally known, or, as far as we are aware, has been translated from the Italian, is the treatise on the poison of the viper, to which his researches in structural anatomy form a kind of appendix. This is, indeed, a good book; but those alone which fully represent Fontana's labours and merits as an experimentalist are the several works

* See Gerber's General Anatomy, Appendix, p. 100.

† Experimental Inquiries, &c.—London, 1771-4, and Phil. Trans. vol. lxiii.

which he published on irritability, by which we are sure that a prior claim might be established for him as the author of many discoveries of which his successors have enjoyed the reputation.

With Fontana we may close the history of the first period of the study of structural anatomy by the microscope. Chiefly by the great influence of Haller, who was but a cold friend of microscopic examinations, the minds of physiologists were now turned to an almost exclusive study by experiment; and how completely all confidence in the microscope was lost at the beginning of this century may be judged from the fact, that the researches of which we have just traced the history were almost excluded by Bichat from his system.

The promulgation of Bichat's system is commonly spoken of as the birth-time of structural anatomy. But this is not strictly true. The origin of structural anatomy was, as we have already said, at least contemporaneous with that of descriptive anatomy; and for three centuries before the time of Bichat it had made a slow but steady progress. He did but add a vast amount of facts, systematize all that had been accumulated, and render all attractive by a physiological theory as fascinating as it was false: in a word, he completed, out of structural anatomy, that which had been before but a partial system of general anatomy. Not that Bichat's merit in general anatomy has been hitherto rivalled; on the contrary, so far as a system could be founded on the structure and properties discernible by the unaided senses, his was perfect; and for nearly twenty years afterwards all who wrote on the same subjects did but condense or comment on his writings. Nay, more, (and this is an unanswerable argument against those who pretend that all investigation of structure is futile which is not microscopic,) Bichat's arrangement of the tissues still stands good; and the microscope has ratified the general correctness of the divisions of tissues which he founded on their obvious and general characters. The truth is, that in the tissues, as in all other objects in nature, where there is a similarity in minute structure there is also a general resemblance. Just as the botanist, for example, can discern at a glance by its general aspect the species or variety of each of the minutest mosses or confervæ, and needs not, except as an ultimate test in difficult cases, to examine the minute arrangement of their parts; so in anatomy, each tissue has an obvious aspect, a gross physical constitution and distinct physiological properties, as well as a certain minute structure, and, for all practical purposes, can be distinguished by the one as well as by the other. In some cases, indeed, these coarser characters can be used for means of diagnosis where the microscope finds nearly identity; as, for instance, between the contractile and non-contractile fibro-cellular tissues, in the cellular tissue in all its varieties, and between the periosteum, and all other (so-called) fibrous tissues.

Upon this truth, we say, it is that Bichat's system still stands almost unaltered, and that much of his labour will still have a place in every prudently-written work on general anatomy. And therefore, although he was no microscopic observer, he contributed a great boon to microscopic anatomy, by showing how animal structure might be studied in an orderly and scientific scheme, instead of the hap-hazard manner in which they were investigated by Leeuwenhoeck and all his imitators.

Many years, however, elapsed before the microscope was again carefully or extensively employed in structural anatomy. Among the first who used it was Sir Everard Home, with the assistance of Mr. Bauer. But to Sir Everard no more praise can be assigned for this than for any other part which he bears in the history of physiology. It does not appear that he was the author of one clear or correct description; and though many of his mistakes may be ascribed to his heedless mode of preparing his objects for examination, yet one is compelled to suspect something more than unintentional error in his accounts, when they cannot by the same careless examination be confirmed; and still more when, in all the investigations which the same Mr. Bauer carried on under the superintendence of Robert Brown, (those, for example, on the fungi and insects infecting corn,) the accuracy of his delineations has never been surpassed.

Others of the earliest microscopic physiologists of this second period were Rudolphi, and Prevost and Dumas; but the first to whom an important place can be given is Gottfried Reinhold Treviranus, one of the most intellectual physiologists of this century, who first in 1816 (in his Vermischte Schriften,) endeavoured, with a philosophic view, to unravel all the tissues into their minutest structures. From this time, and with a regularly-accelerated velocity, the microscope has made progress in anatomy; till now, the rate of discovery is scarcely measurable, and the mind can hardly apprehend the greatness and variety of the results which it each day presents. But at this period of the history we may stop; for most of that which is in this regard important may be with some pains traced in the Report by Mr. Paget, which was published in the last Number. A better task will be to point out by the light of the past history what should be the spirit and tendency of future researches; for at the same time that we thoroughly admire the industry and honest emulation in which the microscopic investigations of the last period have been carried on, and rejoice in the accession to medical science of their brilliant and important results, we cannot help observing that there is now a tendency to error in the entire confidence which many give to statements drawn from microscopic evidence, and in the exclusiveness with which many rely upon the microscope, as if it were the only source of learning now left us for our use, or as if the facts it yields need only be collected in order that physiology may be perfected.

III. The fate of Leeuwenhoeck's discoveries, compared with that of Malpighi's plainly enough showed what every succeeding period has confirmed, namely, that facts of minute anatomy are useless for the advance of science unless they are at once, with a right judgment of their import and their relations, set in the place which they should occupy in the current physiology. No example can show more plainly than Leeuwenhoeck's that labour can make a discoverer, but can never, alone, make a philosopher; and that a microscope, however good and however well used, can never make a physiologist. With all his knowledge of facts, Leeuwenhoeck was neither an anatomist nor a botanist, nor, in a word, anything but a mere microscopist; and the same may be said of Ledermüller, Della Torre, and a number of his less eminent imitators. On the other hand, we see by the example of Malpighi of how great avail the microscope may be made, even in its less perfect state. But it requires a physiologist to interpret its facts for physiology, a botanist to make good use of its results for botany: each science in which it can be employed must use it as an assistant, and in subordination to the laws and the facts obtained by other means; it cannot, in any science, take peculiar preeminence, much less can it in any accomplish so much that either thought or any other method of investigation can be dispensed with.

In the present day there are many Leeuwenhoecks in desire, if not in fact: that is, there are many who think their time well occupied in seeing, without understanding or explaining, the various objects of microscopic investigation. They are unknown in the world of science, because they do not, like their prototype, stand nearly alone; and one can only regret that so much time and labour of industrious, though not brilliant men should be lost. In the same spirit which actuates them separately the Microscopic Society has been founded. A number of persons—anatomists, botanists, geologists, chemists—are here combined, as if to form one large and complex Leeuwenhoeck, with nothing more in common in their disorderly pursuits than that they all study little things with the same apparatus. With as much propriety might a society be formed of those who will examine none but large things; for although the improvement of the microscope be stated as one of the purposes of this association, we cannot find by their proceedings that they have in the smallest degree contributed to this object. We have no doubt whatever that this society is even mischievous, by giving to an apparatus the highest place in the investigation of knowledge; and by inducing industrious men to study little pieces of every science, because they have the means of *seeing* the objects concerned in them. It is in this way that the microscope may be again, as it once already has been, employed to no good purpose. The truth is, the microscopic society exactly fits the foolish spirit of these times, in which men seem to think that a society and transactions are essential to the progress of every artificial division of science; and to compass the formation of a society, will take anything for a bond of union.

There is another reason also why the spirit of the times is peculiarly favorable to microscopic investigations, namely, because their fruits are mere facts, and often very small and unimportant ones. Now, since in the last Number we paid a very matter-of-fact compliment to this spirit by giving a large space to facts in their most condensed and unalleviated form, it must not be thought amiss if, in the present, we protest somewhat against the influence which those derived from the microscope are allowed to exercise; for we hold that facts never yet made a science, and especially that microscopic facts and those which divide the fashion with them—statistical facts—will never make a true physiology. They may grow in size and number, but such facts never improve in nature; they have, if we may so speak, no vital principle, no *nisus formativus* in them; they increase by super-position into a huge amorphous mass; but they never, by any force of their own, shape themselves into the symmetry of a science. For this they must be subjected to the formative influence of the understanding, which cannot use them rightly unless it have been better educated and exercised than in these times it usually is. For in the present day, men, thinking it enough if they pursue and discover facts, and exercising those lower faculties of the mind which can apprehend isolated truths, are too apt to neglect the cultivation of their nobler law-discerning powers.

It is certain that, in proportion to the labour bestowed upon it, the real progress of physiology through microscopic observation, great as it is, has, on the whole, been slow. If, for example, we compare the advancement of the knowledge of the physiology of the nervous system with that of the circulation or secretion or any function of which the apparatus is clear to the assisted sight, we may at once discern the inferior value of mere observation. And it may be safely predicted that if the present love for isolated facts continue long, the progress of the higher truths of physiology will be still slower: for men are already educated for the study of facts rather than for the improvement of the thinking faculty; and with each succeeding year the number of soundly-thinking men must be diminished. It is strange indeed to see how men's minds are bent from that to which they were adapted by nature, by the fashion of the times they live in. Already we can see many suffering their highest faculties to lie in idleness, while they betake themselves to mere looking, and descend to the common low-level of the day; falling under the spell of fashion—becoming microscopists when they might and should be physiologists.

Another circumstance well calculated to make microscopic researches popular in the present day is, that much of the responsibility for accuracy in making them is thrown from the observer upon the apparatus; and that though much labour is requisite for success, it is, for the most part, labour of the dull, mechanical kind, for which energy and intellect are hardly necessary, if, indeed, they can be employed in it. This is truly the age for the glorification of apparatus; magnifying and counting instruments seem to be regarded by many as if they were fitted to do the work of the reason and the understanding; and we fear that the assertion of one of the best German anatomists, that "the future progress of physiology depends upon Schiek and Pistor, the microscope-makers," is but the expression of the general feeling of the majority of those who are pursuing medical science; for a man is now-a-days looked on coldly who advances an opinion which is not made to seem true by a great array of figures of calculation or of measurement.

As a proof that physiology, under the influence of this love of facts, may be studied too exclusively by the microscope, it should be remembered that there are several instances already detected in which similarity of microscopic structure is by no means safe evidence of a similarity of function. An epithelium-cell is very like a gland-cell, a hair-fibre like a smooth muscular fibre, and the physiologically different varieties of fibro-cellular tissue are not discernibly different in structure. These, then, are clearly subjects which can be completely or safely investigated only by experiment or by some of those other methods which the strict adherents of the microscope would throw into disrepute.

Again, at the very best, the microscope teaches only the coarse outlines of the forms of the apparatus in which the processes of the living body are carried on; and the apparatus which we thus see are not the parts by the mutual influences of which the processes are effected. These processes depend, without doubt, upon the mutual relations of the elementary particles, the atoms of the body; and the distance by which we are by the microscope brought nearer to these is probably in comparison with the distance at which they are still removed from our view, infinitely small. The best knowledge, therefore, which the microscope can afford may be compared with the kind of acquaintance with chemical science which a man might acquire from an examination of the apparatus in a laboratory. In thinking otherwise men confound, as they are very apt to do, relative smallness with absolute simplicity; whereas, in truth, there is no reason to believe that the processes effected in a single organic cell are in their essential nature less complex or less obscure than those which go on in a large mass, or in a whole organ, and which are often only the same processes on a yet larger scale.

To whatever extent, then, microscopic investigations may be carried, and whatever improvements may be effected in the instrument, physiology, we think, will make but slow progress if all other means of study be not as diligently employed as if no microscope existed; and, above all, if the understanding, well trained and exercised, be not continually exerted to give form and vitality to the facts which the senses can discern.

In maintaining this we are far from desirous of seeing the ideal systems pursued by such men as Dr. Klencke gain ground in England. Nothing can show better how futile they are for advancing physiology than the history of general anatomy which we have just traced. For we see in it, as in the history of many other sciences, that, after making a certain progress in direct proportion to the industry with which the senses and the understanding were exercised upon it, it was arrested till an art enabled men to employ, not their reason, but their senses, with a supernatural force. At the time of Bichât a full measure of facts had been filled up; the unaided senses had perceived nearly all that lay within their range; and the understanding, by a fair induction, had discerned many laws. After this time there was, as we have shown, a pause for nearly twenty years; a period of calmness, in which the reason, placed as it was upon a high eminence of facts, and undisturbed, might have discerned (had it been sufficient, as idealists imagine, for the unveiling of the great truths of physical science,) many things that were suprasensuous and many still hidden laws. But the reason was employed in vain; and not one step did the science of histology advance till men again resorted to the microscope to assist their senses. If truth, therefore, of any sort be the object of pursuit, we have an unanswerable argument against idealism in the comparison of the results obtained in the last ten years of microscopic observation, with those of the preceding twenty years of meditation. With every inclination which self-love can excite in favour of the belief that man can think the truths of physical science, it is impossible to resist the fact that in this, as in so many other instances, the truths and laws have been discovered by the senses and the understanding, and by them alone. In general anatomy there is not one good argument for making idealism the rival of induction.

We believe, then, that a love of mere facts and idealism, or the love of exercising the imagination in the delusion that the reason is being employed, are the two evil spirits of the day, by which the progress of physiology is retarded. Both work with the highest of their power in Germany, and they are maintained there by their mutual opposition. Both, too, are alike opposed to induction: which, as it is of English origin, ought to become each day more characteristic of English physiology. Microscopists are constantly saying that the microscope is to them what the telescope is to the astronomer. We wish they would make it so: if they would learn how much of astronomy is a mere matter of assisted sight, and how much is composed of laws worked out by the understanding, they could not but be led to make a great change in their mode of study. If astronomers had been merely telescopists, astronomy, properly so called, had certainly never existed: by the same rule, a century of Leeuwenhoecks would never have made a physiology, nor would all their senses have discerned so much as did the understanding of John Hunter.

We believe that if the understanding be exercised with an energy proportioned to the industry with which facts are pursued, the present will be a more brilliant period in the history of physiology than ever yet was known; for never were so many engaged in the pursuit, and never was there so much labour bestowed upon it; and already, by the few who combine clearness of thinking with accuracy of observation, some most striking and important results have been attained. Only, when we see an apparatus exalted so much above its due state of subserviency, we cannot help fearing lest much of that which is being done should be done in vain; and lest that which is gathered in disorder, and often with a heedless curiosity, should end, as it did once before, in mere obscurity.

---

